# WheatSpikeNet: an improved wheat spike segmentation model for accurate estimation from field imaging

**DOI:** 10.3389/fpls.2023.1226190

**Published:** 2023-08-25

**Authors:** M. A. Batin, Muhaiminul Islam, Md Mehedi Hasan, AKM Azad, Salem A. Alyami, Md Azam Hossain, Stanley J. Miklavcic

**Affiliations:** ^1^ Department of Robotics and Mechatronics Engineering, University of Dhaka, Dhaka, Bangladesh; ^2^ Department of Mathematics and Statistics, College of Science, Imam Mohammad Ibn Saud Islamic University, Riyadh, Saudi Arabia; ^3^ Department of Computer Science and Engineering, Islamic University of Technology, Gazipur, Bangladesh; ^4^ Phenomics and Bioinformatics Research Centre, University of South Australia, Adelaide, SA, Australia

**Keywords:** plant phenotyping, wheat spikes, cascade RCNN, segmentation, deformable convolution network

## Abstract

Phenotyping is used in plant breeding to identify genotypes with desirable characteristics, such as drought tolerance, disease resistance, and high-yield potentials. It may also be used to evaluate the effect of environmental circumstances, such as drought, heat, and salt, on plant growth and development. Wheat spike density measure is one of the most important agronomic factors relating to wheat phenotyping. Nonetheless, due to the diversity of wheat field environments, fast and accurate identification for counting wheat spikes remains one of the challenges. This study proposes a meticulously curated and annotated dataset, named as SPIKE-segm, taken from the publicly accessible SPIKE dataset, and an optimal instance segmentation approach named as WheatSpikeNet for segmenting and counting wheat spikes from field imagery. The proposed method is based on the well-known Cascade Mask RCNN architecture with model enhancements and hyperparameter tuning to provide state-of-the-art detection and segmentation performance. A comprehensive ablation analysis incorporating many architectural components of the model was performed to determine the most efficient version. In addition, the model’s hyperparameters were fine-tuned by conducting several empirical tests. ResNet50 with Deformable Convolution Network (DCN) as the backbone architecture for feature extraction, Generic RoI Extractor (GRoIE) for RoI pooling, and Side Aware Boundary Localization (SABL) for wheat spike localization comprises the final instance segmentation model. With bbox and mask mean average precision (mAP) scores of 0.9303 and 0.9416, respectively, on the test set, the proposed model achieved superior performance on the challenging SPIKE datasets. Furthermore, in comparison with other existing state-of-the-art methods, the proposed model achieved up to a 0.41% improvement of mAP in spike detection and a significant improvement of 3.46% of mAP in the segmentation tasks that will lead us to an appropriate yield estimation from wheat plants.

## 1 Introduction

Wheat is one of the top three most valuable crop species around the world, as well as responsible for nearly half a billion dollars’ worth of annual trade (Hasan et al., 2018). It is the principal source of nutrition for 2.5 billion people in 89 different nations (Misra et al., 2020). As the global population rises, so does the need for cereal grains including wheat, sorghum, millet, maize, and rice. This is because these grains provide sustenance for a sizable population around the world. Wheat accounts for the vast majority of the world’s food commerce and is cultivated on more land than most other crops. For good reason, wheat is often referred to as the foundation of food security (Xiong et al., 2019). This highlights the need to identify wheat plant varieties with greater resilience, higher yields, and enhanced endurance to biotic and abiotic stresses.

**Figure 1:**
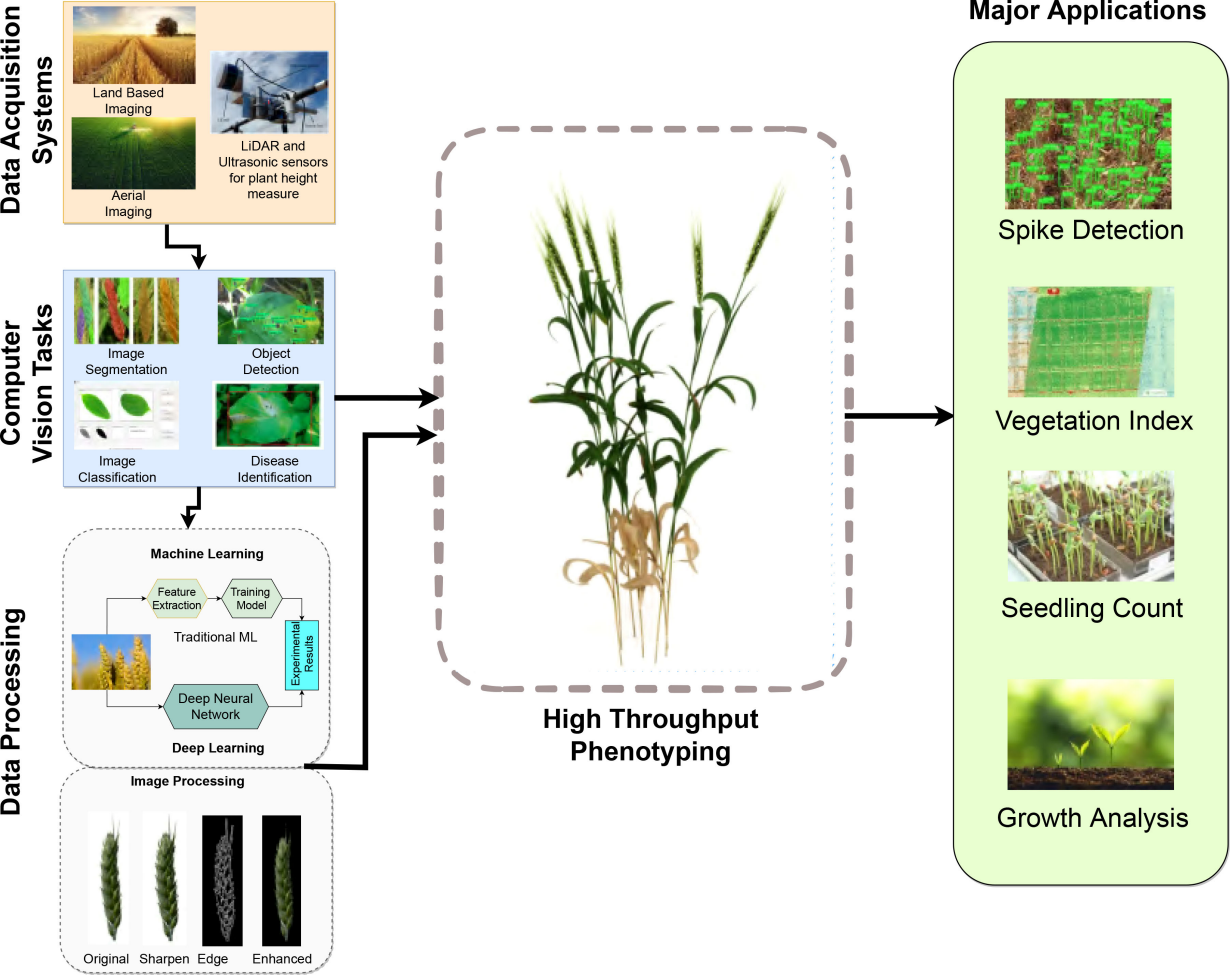
Overall high throughput phenotyping process for various domain applications.

In agriculture, scientific efforts are directed toward quantifying how a plant’s function and features are continuously affected by its environment. While these tasks of plant and crop phenotyping are not new, manually keeping track of a plant’s physical and biological attributes such as its height, growth rate, hardiness, nutritional content, and yield, as a function of environmental conditions can be extremely time and labor demanding. Modern high throughput plant and crop phenotyping methods, on the other hand, involving image-based information, captured by configurable land-based equipment or drones with minimal effort and mostly with little expense, are able to collect significantly more data in a considerably shorter length of time. The challenge now is to effectively and efficiently process this vast amount of data, and derive as accurate information as possible about plant growth and development in an automatic way and in a practical period of time. To meet this challenge cutting-edge agricultural technology is now making use of *artificial intelligence* (AI) in order to greatly simplify the modern crop performance monitoring process overall. The complete processing chain of steps involved in modern high throughput plant phenotyping operations, depicted in **Figure 1**, may thus include ground or aerial imaging, or both, the application of image processing methods of computer vision and the application of machine learning processes (*i.e.*, AI) for, say, object recognition, object identification and instance segmentation. This information is then used as input into subsequent data analysis procedures where correlations with genetic and environmental factors are deduced.

Accessing image-based phenotypic features such as wheat crop yield, and even wheat spike size and shape, requires accurate segmentation of wheat spikes out of a complicated backdrop. When it comes to wheat spike segmentation, several different kinds of *convoluted neural network* (CNN) analysis systems have been generated including, but not limited to, Faster R-CNN, Cascade R-CNN, Mask R-CNN (Ren et al., 2015; He et al., 2017; Cai and Vasconcelos, 2019). These deep learning algorithms have been trained on massive spike data sets so that they will subsequently be able to distinguish spikes from other object features (background) in images. The insights gained might then be put to use in real-time for data intelligence, predictive analysis, and smart farming, all driven by artificial intelligence. Earlier, the author Batin et al. (Batin et al., 2023) proposed improved detection of wheat spikes using multi-stage CNN; this time, author significantly improves both the detection and segmentation process of wheat spikes, leading us to a more accurate spike estimation approach that is robust in terms of identifying it in the complex in-field scenarios. The objective of this research is to achieve greater accuracy and consistency than previously proposed deep learning techniques, utilizing a method that can segment wheat spikes from images of actual fields, and then to apply this strategy to other agricultural sectors where AI can be advantageous.

The challenge of segmenting objects in complicated contexts may be overcome, in part, by developing multi-stage, *region-based convolutional neural networks* (RCNNs), which employ region suggestions in conjunction with CNN characteristics (Maji et al., 2022). When the calculated CNN-derived features obtained from region proposals are used, segmentation as well as counting of objects in pictures is superior to approaches that do not use region-based proposals.

In this study, we aim to address the phenotyping challenge of accurately segmenting wheat spikes from real-world field images with high levels of precision and robustness. The ability to precisely detect, count and segment wheat spikes from such images is crucial for accurately estimating the overall wheat yield, more so importantly in a non-destructive manner. The focus of this research problem is the development of advanced deep learning based approaches that may successfully manage the complexities and variances present in such images. These complexities arise from things like varying illumination, occlusions, and various growth stages of wheat. As can be seen from Section 2, existing research in non-destructive phenotyping mostly focuses on lab condition images, which cannot always represent the real-world complex scenarios.

To meet the requirements of our study, we have first curated and annotated a wheat spike segmentation dataset, *SPIKE-segm*, with accurate masks and bounding boxes. We then proposed a modified spike segmentation model based on the Cascade Mask R-CNN architecture. Our proposed method includes novel modification to several components of the model architecture, including Deformable Convolution Network (DCN), Generic RoI Extractor (GRoIE), and Side Aware Boundary Localization (SABL). Details of these modifications and an ablation analysis between these components have been explained in Section 3 and Section 4. In terms of spike segmentation, based on the results presented in Section 4, we have determined that our proposed method is more accurate, efficient, applicable, and robust than other existing methods (see Section 4). Finally, the conclusions are summarized in Section 5.

## 2 Related works

All facets of modern life are becoming more dependent on technology. The agricultural industry has been profoundly altered by technological advancements. Many researchers in the field of agriculture are utilizing AI to modernize age-old practices, therefore increasing output while decreasing the workload of farm workers and their demands on the environment. Many techniques for phenotyping plants have been reported by scientists in the field. Different deep-learning approaches have been proposed for segmenting and counting wheat spikes.

Misra et al. (Misra et al., 2020) introduced SpikeSegNet, a novel *deep learning* (DL)-based method for the detection, recognition, and counting of many wheat spikes. On average, the suggested technique was 99% precise, 95% accurate, and 97% robust when used to count spikes in images their data set. The SpikeSegNet approach achieved sufficient robustness based on a data set containing illuminated images, with no significant drop in segmentation performance. Despite extensive testing under a variety of lighting conditions, results from the lab are not feasible to apply to real-life scenarios. On the other hand, the proposed method requires sufficiently high-quality images to be able to detect and count spikes.

Zhang et al. (Zhang et al., 2022) suggested a Hybrid Task Cascade model improve detection outcomes for the wheat spike identification problem in high-dimensional environments, making it possible to reliably segment wheat spikes. Wheat spike detection and segmentation in a wheat field with complicated surroundings is the major topic of this study. For the model’s bounding box and mask, values of *average precision* (AP) equal to 0.904% and 0.907% were achieved, respectively, while a value of 99.29% was found for the precision with which wheat spikes might be enumerated. Even though both bounding box and mask segmentation have room for improvement in terms of average precision. An improved performance of Wheat-Net was found in the experiments presented in this study, although when it came to identifying the base of wheat spikes, the model encountered several segmentation issues; due to their similarity in color, texture, and form to the plant background, the connected spike and stem made up a blurry boundary.

The work by Hasan et al. (Hasan et al., 2018) presented a fine-tuned *region-based convolutional neural network* (R-CNN) model for detecting and evaluating wheat spikes in ground-based images. Faster R-CNN was chosen as the network model to be instructed in this article’s training set. Images were fed into a pre-trained VGG-16 prototype, which was subsequently utilized to automatically extract features, and then sent to the *region proposal network* (RPN) to generate *bounding boxes* (Bbox-es), and finally to the classification network to be labelled as spikes or background. In-field images were captured using high-definition RGB cameras to create a spike data set called SPIKE, which was used to train a Faster RCNN architecture. Both the average accuracy and F1 score for the model were 93.4% and 0.95, respectively. Nonetheless, no actual spike segmentation work is carried out here. However, the model’s inaccuracy increased when dealing with partially covered spikes, especially in high-density locations.

A strategy based on SpikeRetinaNet was developed by Wen et al. (Wen et al., 2022) to recognize and quantify a densely distribution of small objects in complicated images. The three main components of SpikeRetinaNet − including the use of BiFPN (weighted *bi-directional feature pyramid network*) for more efficient implementation of multi-scale data, and the use of Soft-NMS (*non-maximum suppression*) to address the occlusion problem − make it an upgraded edition of the RetinaNet framework. The authors trained and evaluated their technique using the GWHD data set, which was supplemented with pictures from the *wheat-wheat grass spike detection* (WSD) dataset. One drawback of this approach is that it may generate many bounding boxes for the same spike. Again, the outcome differs depending on the growth stage of the crops. Despite Mean average accuracy (mAP) rates for wheat spike identification achieved by the model were 92.62%, with a count detection capacity of 92.88%.

For segmenting individual corn against a ground background, Jin et al. (Jin et al., 2018) suggested a combination of deep learning and regional development techniques to identify the root of specific maize plants in a variety of settings. For target detection, Faster R-CNN was used because of its particular search with a *regional proposal network* (RPN). A faster R-CNN-based technique was found to be effective at recognizing stem anchors in 2D views from 3D Lidar pictures. The model has a drawback in that if the stem is totally missing in the scanned data, the system will not be able to recognize the individual maize plant, which will not be able to develop into an individual stem. The suggested approach was only tested on premature maize plants, and its efficacy for segmenting adult maize plants requires more study.

Zhou et al. (Zhou et al., 2018) proposed a new approach that uses computer vision to obtain statistics on the number of wheat spikes. Their effort presents a technique for improving the maximum entropy segmentation technique by selecting optimal thresholds for noise reduction using morphological filters, which yields more reliable coarse-segmentation findings. Multispectral and panchoromatic pictures are fused in this case. These findings not only demonstrate the high precision of the approach used here, but also demonstrate how the local search operator may greatly enhance the performance of the original evolution algorithm, which has its own inherent limitations.

Su et al. (Su et al., 2020) suggested Mask R-CNN for the reliable identification of disease sites and symptom severity on wheat spikes. The fungal disease *fusarium head blight* (FHB) causes significant losses in quantity and quality of wheat grains. To build the feature pyramid and to extract features, Mask-RCNN relied on a network similar to ResNet-101 called the *feature pyramid network* (FPN). After full-sized image of wheat spikes were used to create mask images, Mask-RCNN was used to forecast unhealthy spots on each spike. Despite the bigger dataset, naturally infected wheat spikes were left out, and the dataset was collected under controlled environmental settings. The detection rates for wheat spikes using this procedure were 77.76%, and for infected regions, they were 98.81%. By comparing the predicted wheat FHB severity value to the actual value, they were able to attain an accuracy of 77.19% in their predictions.

Most spike segmentation approaches have only been evaluated on random data sets in the lab, making it hard to determine whether or not they will provide correct findings when applied to real-world images. The images were taken in controlled settings, resulting in good resolution and recognizable spikes; the number of obscured spikes is very minimal. As a result, the techniques often perform poorly under real-world imaging conditions when only partially visible spikes are present, particularly when the resolution is low. In contrast, we evaluated our model (described in the next section) on low-resolution, real-world image data and found an increased capacity to segment (and count) partially hidden spikes.

## 3 Materials and methods

**Figure 2:**
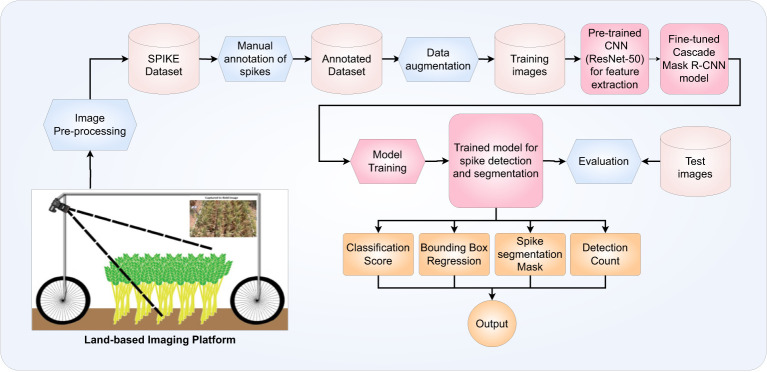
Generic work flow diagram describing the proposed algorithm for the detection and segmentation of wheat spikes in field images.

The objective of our efforts is the development of a faster, more robust and more accurate method of identifying and counting wheat spikes in land-based field images. A flow-chart diagram describing the process involved in our system is shown in **Figure 2**

### 3.1 Data collection

For this research, we have utilized the SPIKE data set that was established by Hasan et al. (Hasan et al., 2018). The SPIKE data set was collected over four months (July 21, 2017 - November 22, 2017) using a transportable, land-based imaging frame, taking images of 90 individual plots of wheat distributed over 18 rows and 5 columns. Ten different varieties of spring wheat (*Triticum aestivum L.*): Drysdale, Excalibur, Gladius, Gregory, Kukri, Mace, Magenta, RAC875, Scout, and Yitpi, were cultivated in the areas. Having spikes of varying shapes and sizes increases the versatility of the data set for the task of recognizing different wheat varieties (not of a focus of this work). Three fertilizer treatments were applied to each variety of wheat: no fertilizer treatment, early treatment, and late treatment, in order to establish the effect of fertilizer on wheat spike development. Each variety and each different treatment was replicated three times for a statistical analysis. Two-thirds of the plots were given the industry-standard fertilizer dose of 80 kilograms of nitrogen, 40 kilograms of phosphorus, and 40 kilograms of potassium per hectare, whereas the remaining thirty plots were left untreated.

**Figure 3:**
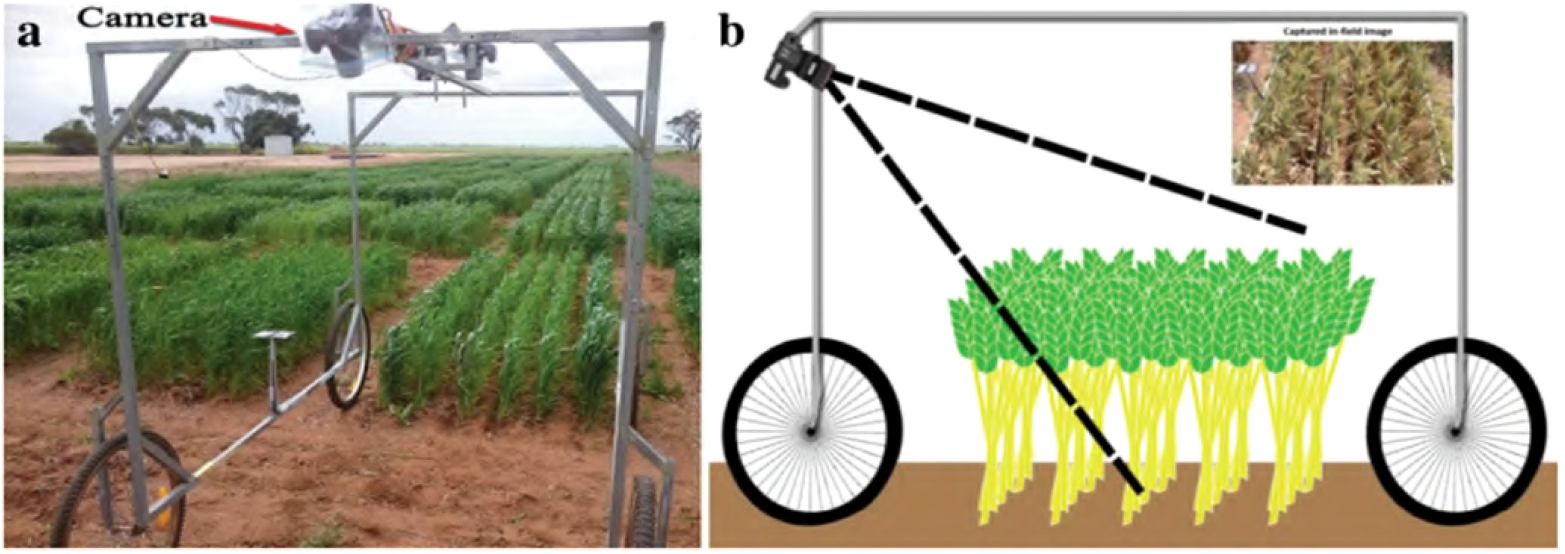
The ground-based vehicle for imaging in the field. **(A)** A camera, angled for oblique viewing, is placed at the top of an imaging frame mounted on a four-wheel base (the wagon). The frame also supports two stereo cameras, angled vertically, placed in the center of the top section. **(B)** A schematic of the wagon from a side-view (Hasan et al., 2018).

Three cameras were mounted on an overhead rail in the center of a steel-framed, four-wheeled cart shown in **Figure 3**. Although the setup includes a stereo pair of cameras for “overhead” observation, the images from these cameras were not included in this study (spikes seen along the vertical viewing axis appear small and round in photos, finding them challenging to identify). Instead, only images taken by a camera situated at one end of the cart, supported at an acute angle to the vertical were used in this study. The images were taken with a digital camera with a resolution of 18.2 megapixels using a Canon EOS 60D. After a trial and error period, it was determined that a viewing angle of 55 degrees from the horizontal, overhead rail would provide the most usable plot area with the least amount of intersection. The height of the camera’s sensor from the ground was 190 centimeters. These are the parameters that had been set for the camera;

- Focal length —18 mm,
- Aperture — f/9.0,
- ISO — automatic
- Exposure time — 1/500 s

### 3.2 Dataset annotation and preparation

For the segmentation of wheat spikes from field images, we manually annotated images from the SPIKE data set to create training, testing and validation sets. The images in this data set are divided into the following three color classes: GSYC - green spike, yellow canopy; GSGC - green spike, green canopy; YSYC - yellow spike, yellow canopy. We maintained this same distribution of images, which correspond to the different growth stages of wheat. (**Table 1**) shows the overall count and distribution of the data set for wheat spike segmentation:

**Table 1:** Number of images from each growth stage used for training, testing and validation.

Images	GSYC	GSGC	YSYC	Total
**Train**	222	34	34	290
**Val**	9	3	3	15
**Test**	9	3	3	15
**Total**	240	40	40	320

**Figure 4:**
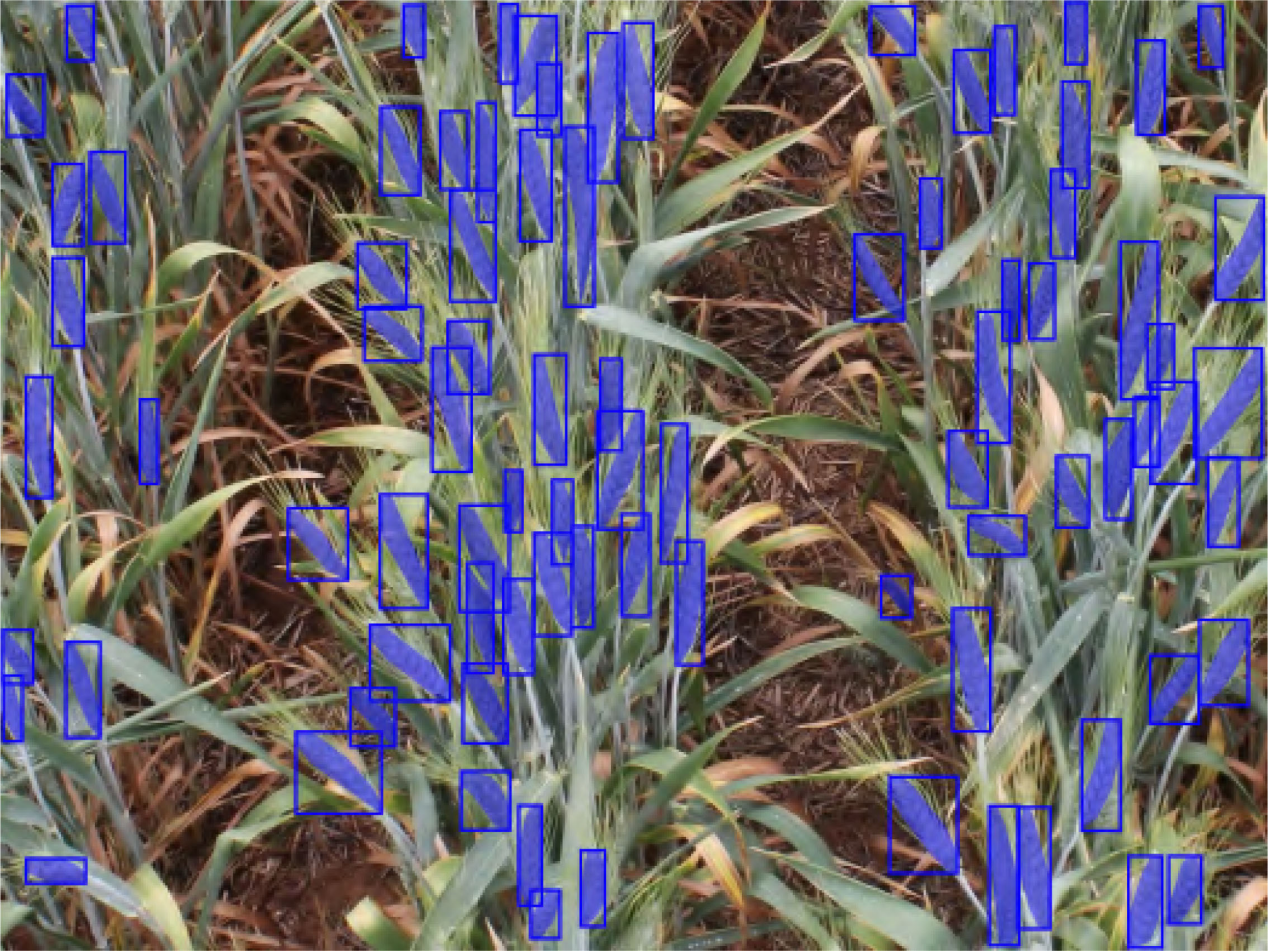
Example of the annotation of an image from the SPIKE data set (GSGC stage).

To annotate the images in the data set we used the Roboflow Web API (Dwyer et al., 2022). Wheat spikes in the images were annotated using the polygon tool provided by API. Careful inspection ensured a high quality of annotations, as exemplified by **Figure 4**. More than 26,000 spikes were annotated with an average of 83 spikes per plot image. Considerable effort went into curating this spike segmentation data set. For evaluation and testing purposes, we annotated the objects (wheat spikes) in the images in the COCO format (Lin et al., 2014), which is used as a standard format to evaluate instance segmentation models.

### 3.3 Proposed wheat spike segmentation approach

#### 3.3.1 Model architecture

To detect, segment and count wheat spikes from images, we decided the *instance segmentation method* to be the most suitable for the task at hand. The high resolution of the plot images, allowed the segmentation model to detect, localize and segment spikes from features at the instance level and thus be able to count the detected spikes as well. We use the Cascade Mask R-CNN model, a two-stage object detection and segmentation approach described by Cai et al. (Cai and Vasconcelos, 2019), for segmentation. This method is a multi-stage, modification of the Mask R-CNN architecture (He et al., 2017) allowing the detectors further down the cascade architecture to be successively more discriminating against false positive detection. These steps of the R-CNN architecture are trained progressively, utilizing the output of the preceding stage to train the subsequent stage.

**Figure 5:**
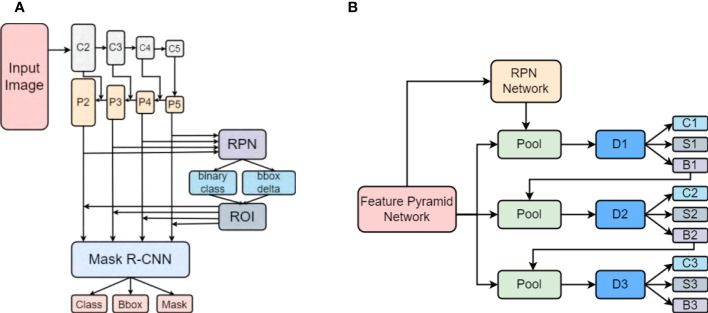
The different architectures of **(A)** Mask R-CNN and **(B)** Cascade Mask R-CNN (Cai and Vasconcelos, 2019).

**Figure 6:**
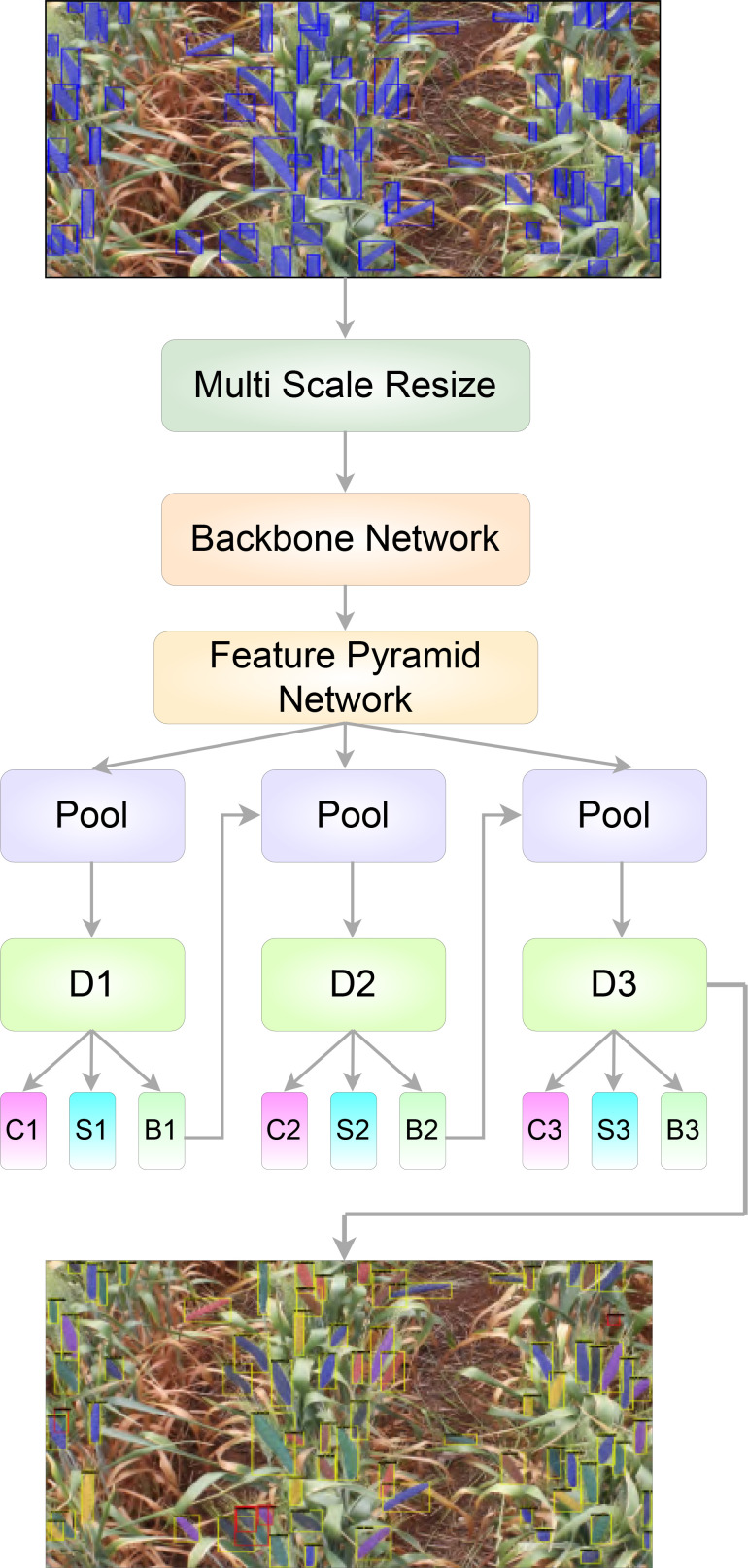
The proposed model structure for spike segmentation.

By training a segmentation branch in tandem with a detection branch, Mask R-CNN effectively expands upon the two-stage design of Faster R-CNN (Ren et al., 2015; Lin et al., 2017) used by Hasan et al. (Hasan et al., 2018). **Figure 5** depicts the architectural representations of Mask RCNN and Cascade Mask RCNN. Here, I - input image, conv - convolutional layer, pool - maxpooling, and C, S, B represents classification, segmentation and bounding box head, respectively. In comparison with Mask RCNN (**Figure 5A**), the Cascade Mask RCNN (**Figure 5B**) has multiple detection branches, which raises the question of how many and where to add segmentation branches. For our project we opted for a design in which a segmentation branch is included at each cascade stage. At the time of inference, the final mask prediction for this architecture is derived from the ensemble of three segmentation branches. The overall structure of our model is shown in **Figure 6**.

#### 3.3.2 Model optimization

**Figure 7:**
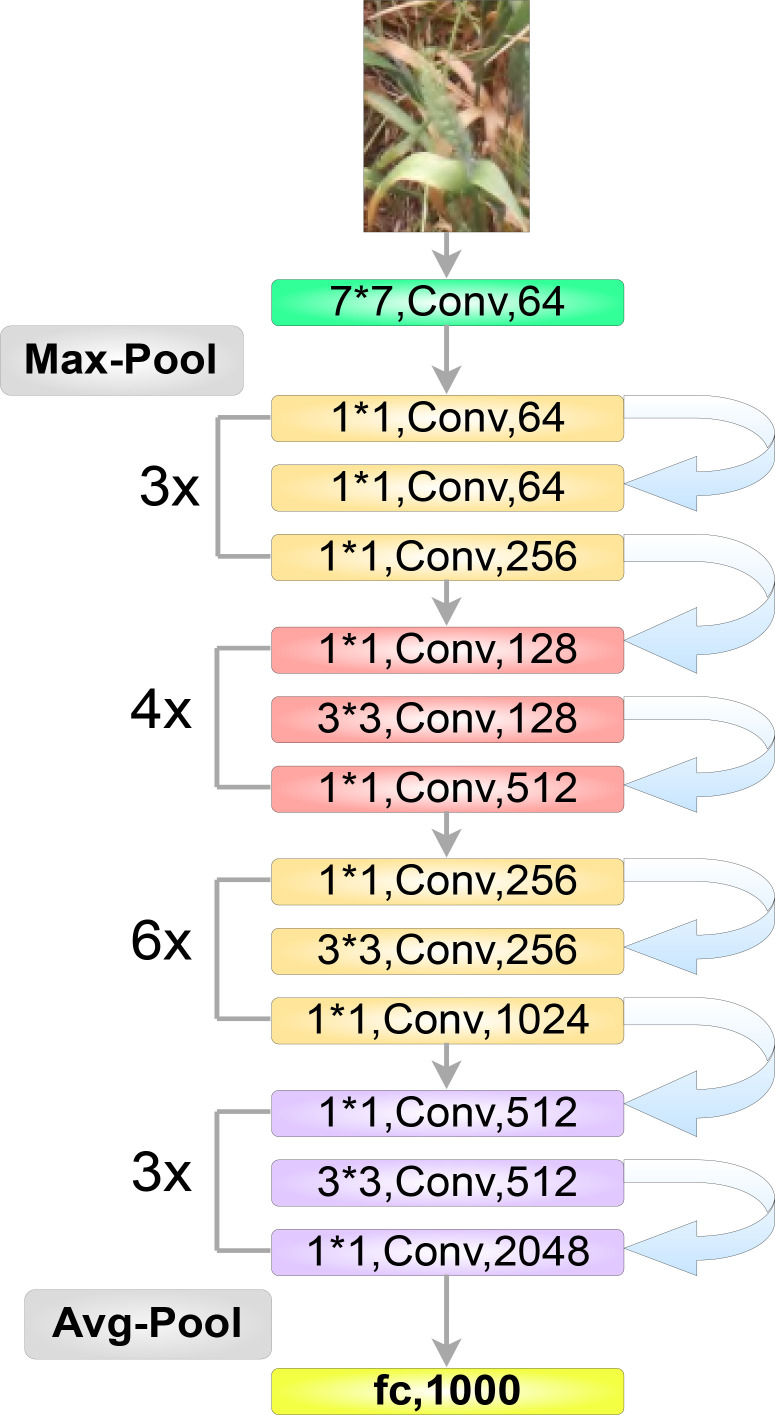
The optimized ResNet50 architecture highlighting multiple blocks, each comprising three convolution layers.

The first step to detecting and localizing objects in an image is to extract relevant features from that image using a backbone network (**Figure 7**). In our study we employed the ResNet architecture (He et al., 2016) as our model’s backbone, which comprises four stages or residual layers wherein, each stage has a different number of convolution “blocks”; in our case: (3, 4, 6, 3) (**Figure 7**). Furthermore, each block has three convolution layers, and each convolution layer is followed by a batch normalization layer. For our experimentation, we used the ResNet-50 version of the ResNet architecture. It is important for our proposed model to be able to extract relevant features of wheat spikes across the whole input image.

**Figure 8:**
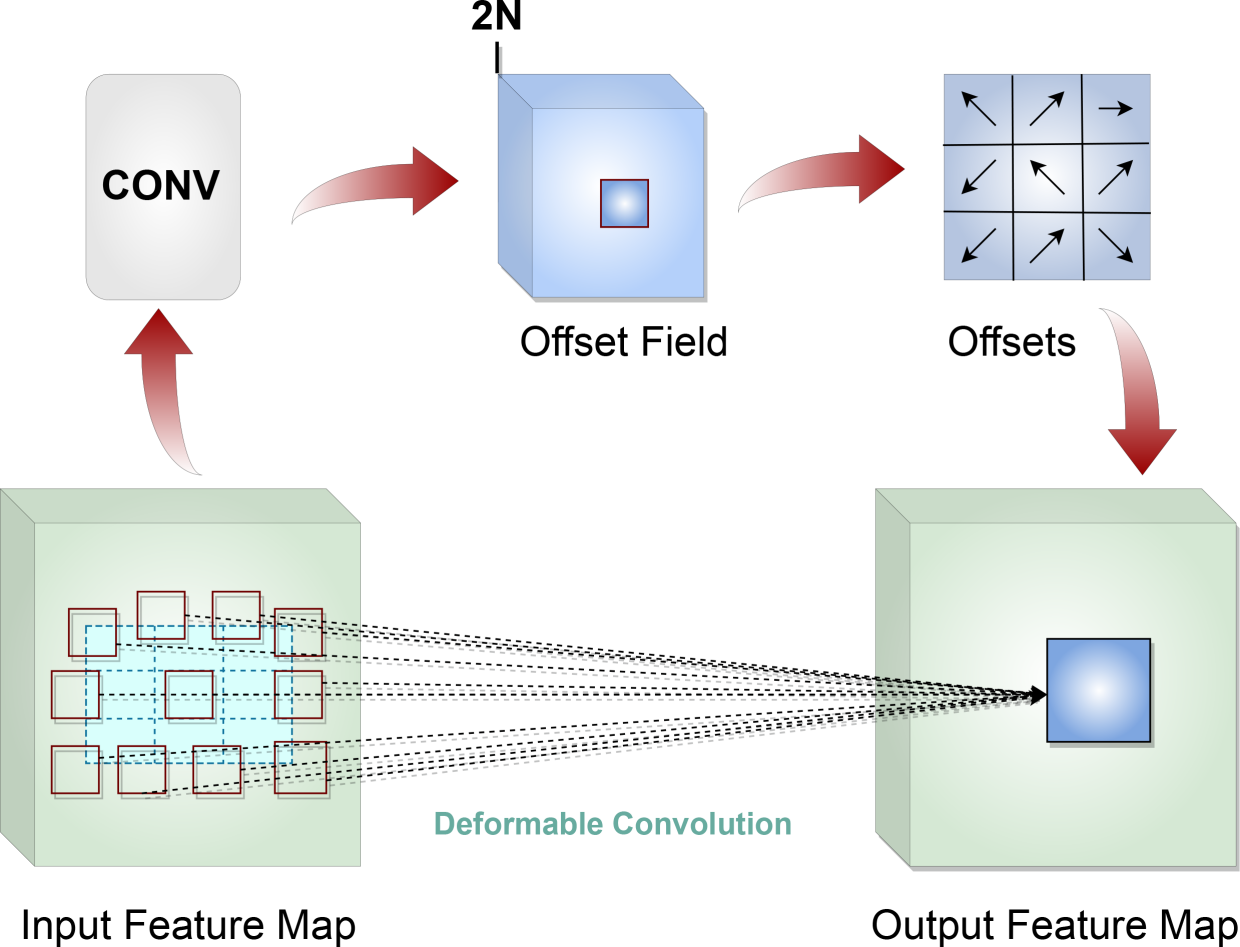
Deformable convolution network.

Due to a high variability in shape and size of wheat spikes, standard convolution layers do not provide the optimal solution, as the fixed size of the kernels in those layers all have the same receptive field (sampling grid) to model geometric transformations of objects in images. The *deformable convolution network* (DCN) (Dai et al., 2017) addresses this issue by allowing the convolution layers of the network to have a deformable structure for their kernels (**Figure 8**). Offsets of the sampling grid are learned by the model without any additional supervision. This learned “deformation” of the kernel allows the convolution layer to model dense spatial arrangements of objects so that overall the model can detect and segment wheat spikes of different size, shape and orientation.

After extracting features from the backbone network, our model arranged those *region of interest* (ROI) features using a *feature pyramid network* (FPN) (Lin et al., 2017) and *balanced feature pyramid* (BFP) (Pang et al., 2019) sub-network fused together, into a multi-scale pyramid with a top-down architecture comprising 5 different scales, each containing 256 feature channels. These features, generated by the FPN + BFP module, are then passed on to a *region proposal network* (RPN) (Ren et al., 2015), which generates candidate regions that might contain an object. To efficiently generate region proposals (or anchors), RPN predicts the object bounding box and classification score of the object with varying scales and aspect ratios. Instead of a single-level RoI extractor, we used a *generic RoI extractor* (GRoIE) (Rossi et al., 2021), which utilizes all the levels of FPN for RoI pooling to extract 7 × 7 RoI features. GRoIE was chosen over a single RoIE based on the observation that only the best layer from FPN is selected by the RoI extractors currently in use, ignoring potentially useful information in other layers. In order to overcome this restriction, non-local building blocks and attention mechanisms were added to the GRoIE to extract and merge data from all FPN layers, producing a more comprehensive and accurate representation of the wheat spikes (Zhang et al., 2022).

**Figure 9:**
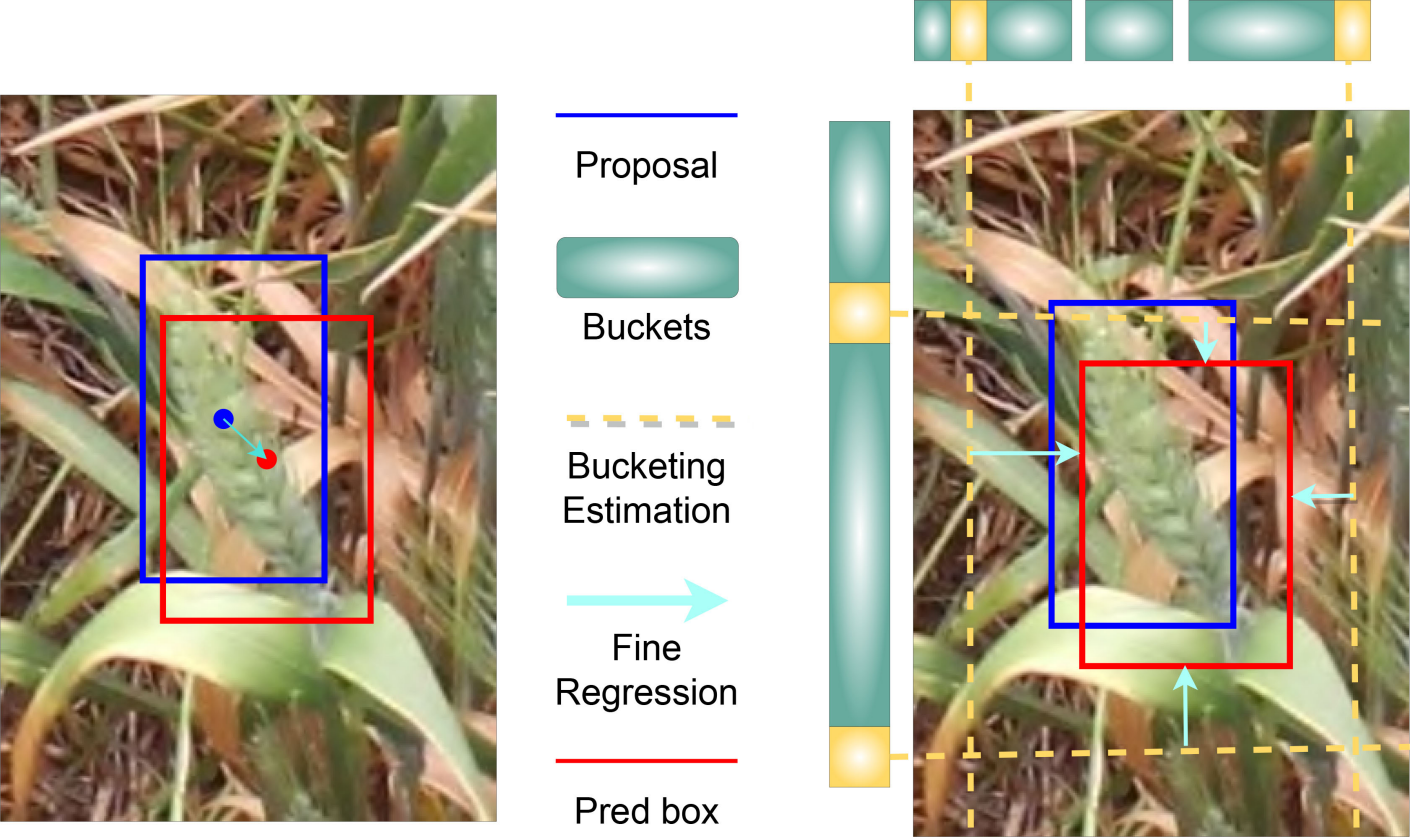
An example of side-aware boundary localization (SABL).

After the RoI pooling, the feature maps were used as input into the cascaded *bounding box* (bbox) head of our model (**Figure 6**). For this bbox head, we used the *side aware boundary localization* (SABL) approach, proposed by Wang et al. (Wang et al., 2020), for localization of each side of a bounding box that might contain a wheat spike. SABL improves the localization performance of the object detection model by focusing on object boundaries rather than on the center point utilized in more traditional bounding box regression schemes. In our approach, side-aware features are extracted from RoI features and then a “bucketing scheme” is employed where the target space (input image) is divided into multiple buckets (shown in **Figure 9**). The bbox head then predicts in a two-step process the bounding box that contains an object - bucket estimation to find a proposed bbox, and fine regression of the proposed bbox.

The model must segment wheat spikes at an instance level after identifying and localizing them. In order to achieve segmentation results, a mask head was added to each cascaded level of the model (**Figure 6**). Again, a single-level RoI extractor was utilized to extract a 14 × 14 RoI feature map used as input for the model’s mask head. The mask head consists of a fully convolutional network (Long et al., 2015) with four convolutional layers and a 3 × 3 kernel.

Deep learning networks need to adaptably fine-tune the *learning rate* (LR). Early model instability can result from a large LR. In our model we used a linear warm-up LR approach (Goyal et al., 2017) for training. The LR is first set to a tiny number, usually a fraction of the real LR, and then progressively increased over a few epochs until it approaches the actual LR. The model starts training with consistent convergence. Our linear warm-up approach starts with 0.01 times the real LR and progressively grows until the actual LR is attained. As the training iteration grows, the LR must decay since a high LR will prevent the model from converging. MultiStepLR, a prominent LR decay approach, employs specified steps (epoch numbers) to reduce the learning rate by 0.5 to address this issue. The MultiStepLR scheduler reduced the LR by 0.5 at epochs 100 and 140 for all models. Even though linear annealing results in a greater *average precision* (AP) for an *intersection over union* (IoU) measure of 0.5, MultiStepLR converges significantly sooner (as shown in the Comparative Analysis section).

Furthermore, we also employed auto-scaling LR based on the batch size and number of images per gpu to obtain the LR for our model (Goyal et al., 2017). According to the linear scaling rule for auto-scaling of LR, if the batch size is multiplied by *k* then LR must also be multiplied by *k*, while other hyper-parameters remain unchanged. The base batch size and LR for the auto scaling rule was set to 16 and 0.01, respectively. So, with a batch size of 2 and 1, the LR was reduced by a factor of 8 and 16, respectively. This is done so that the models’ are not affected negatively by having different batch sizes due to memory constraints. We observed the impact of utilizing the auto-scaling rule in the ablation study part of our experiments. **Table 2** shows the important hyper-parameters that were fine-tuned as part of our method.

**Table 2:** Hyper-parameter table.

Hyper-parameter	Type	Value
Optimizer	SGD	momentum=0.9 weight_decay=0.0001
Batch size		batch_size=1
Learning Rate		Ir = 0.000625
Learning rate policy	MultiStep	step = [100, 140]
Warmup policy	Linear	warmup.iterations=500
		warmup_ratio=0.01
Epochs		Number=150
Classification stages	Cascaded	Number=3
IOU threshold		Stage_1 = 0.5
		Stage_2 = 0.6
		Stage_3 = 0.7
Score threshold	RCNN	Score_thr=0.5

After careful optimization and fine-tuning, we eventually constructed a new instance-segmentation model for wheat spikes based on the Cascade Mask RCNN method, together with ResNet50 backbone with deformable convolutions, generic RoI extractor (GRoIE), side aware boundary localization (SABL) for bounding box regression, and auto-scaling LR rule. During training, several data augmentation treatments were applied, namely, resizing the images to 1333*x*800 resolution, random flipping with a 0.5 probability of being vertical or horizontal, and finally padding the images with up-sampling to a multiple of 32. These data augmentations help enhance the robustness of the model by providing it with more varied training data. The backbone network of the model was initialized with weights from a pre-trained ResNet50 model and parameters of other modules was initialized randomly with different initialization methods such as Kaiming initialization (He et al., 2015) and Xavier initialization (Glorot and Bengio, 2010).

As the model has a cascaded structure with separate bbox and mask heads for detection and segmentation tasks, the overall loss function of the model takes the following form,


(1) $$L=\sum_{t=1}^{T}a^{t}\;(L_{bbox}^{t}+L_{mask}^{t})$$

Here, $L_{bbox}^{t}$ and $L_{mask}^{t}$ are the bounding box prediction losses and the mask prediction losses at different stages t, respectively. Since we are using 3 stages for the cascade architecture, T = 3 and *a* are set to [1,0.5,0.25] for all three stages consecutively.

Smooth L1 loss is used for both region proposals and bounding box regression loss, which can be defined as follows:


(2) $${\text{loss}}_{\text{l}1}{\;}^{{\text{sm}}^{\;}}\;=\{\begin{array}{c} 0.5\cdot \left(\frac{{(|\text{pred}-\text{target}|)}^{2}}{\text{β}}\right)\text{ if }\left|\text{pred}-\text{target}\right|<\beta \\ \left|\text{pred}-\text{target}\right|-0.5\cdot \beta \text{ otherwise} \end{array}$$

Here, *pred* represents the predicted bbox (or region), *target* represents the target bbox (or region), and *β* is the threshold parameter, which was set to 1.0*/*9.0 for the RPN head and 0.1 for all the three bbox heads.

For classifying the bounding box region as well as the pixel in the segmentation mask, Cross Entropy (CE) loss function is used, defined as following:


(3) $${\text{loss}}^{\text{ce}}=\;-\frac{1}{N}\sum_{i=1}^{N}\left[y_{i}\cdot \log(p_{i})+(1-y_{i})\cdot \text{log}(1-p_{i})\right]$$

Here, *y_i_* is the target class and *p_i_* is the predicted class of the bounding box (or pixel), and *N* = 2 as we are only classifying between the *spike* and *background* classes.

#### 3.3.3 Evaluation metrics

For a consistent evaluation of the models we have implemented, we use the *average precision* (AP) metric because of its representativeness and simplicity. AP measures the area under the *Precision-Recall* (P-R) curve, where precision and recall are defined by Equations (4) and (5). Thus, AP can be defined by Equation (6). According to the COCO evaluation protocol, AP can be measured at different thresholds for *intersection over union* (IoU) measures, such as IoU = 0.5 (PASCAL VOC metric), IoU = 0.75 (strict metric), and IoU = 0.5: 0.95: 0.05 (primary challenge metric) (Lin et al., 2014).


(4) $$Precision=\frac{TP}{TP+FP}$$

(5) $$Recall=\frac{TP}{TP+FN}$$

(6) $$AP=\frac{1}{101}\sum_{r_{i}\in \left\{0,0.01,\ldots ,1\right\}}\underset{r_{i}:r_{i}\geq r}{\max}p\left(r_{i}\right)$$

(7) $$IoU\left(A,B\right)=\frac{\left|A\cap^{}B\right|}{\left|A\cup^{}B\right|},$$

where |*S*| denotes the numerical size of the set *S*.

In the above and below, *true positive* (TP) - refers to the case where the model correctly detects a region as a spike, *false positive* (FP) — refers to the case where the model incorrectly detects a background region as a spike, or detects the same spike as multiple ones; and *false negative* (FN) — is where the model incorrectly classifies an actual spike as background. *P* (*r_i_*) is the measured precision at recall *r_i._* The precision at each recall level, *r_i_*, is interpolated by taking the maximum precision-measured for which the corresponding recall exceeds. *A* and *B* represent the predicted and target bbox, respectively, in Equation 7.

#### 3.3.4 Implementation

Training and testing of all the experimental models were done using a Ryzen-5 2600x (6-core) processor, 16GB system RAM and NVIDIA RTX 2070 GPU with 8GB VRAM (unless specified otherwise). We also utilized the open-source object detection and instance segmentation framework MMDetection (Chen et al., 2019) based on the PyTorch deep learning library to implement our model architecture of choice, as it offers an easy-to-use modular codebase. After each epoch of training, we evaluated the model on the validation set. So, after the whole training period, we saved the checkpoint of the best performing model.

## 4 Result

### 4.1 Performance analysis of the model

We report the average precision (AP) of our model on the test set to evaluate its performance. The test set contains 15 images from three different growth stages of wheat and contains a total of 1243 wheat spikes. In our experiment, the object detection score refers to the AP value at a specified threshold, calculated using Equation 6, for the final bounding box prediction stage of our model’s network. Segmentation score refers to the same metric, but for the final segmentation mask output stage. Our model achieved an object detection score of 0.93 and segmentation score of 0.9404 for AP at IoU = 0.5, 0.801 and 0.8018 for AP at IoU= 0.75, and 0.678 and 0.6459 for AP at IoU = [0.5: 0.95: 0.05]. In the case of a dense environment such as a wheat plot, detection models tend to predict many false positives. In our case, the high AP values indicate a relatively low detection rate of false positives.

The detection performance of the model can also be evaluated through the count results on the test set. We set an IoU threshold of 0.5 for a spike prediction to be considered true positive and counted the TP, FP and FN results for each image. **Table 3** shows these count results. As can be seen from the table, our model achieves an average accuracy of 86% and an average F1-score of 0.93, across all 15 test images. This is particularly impressive, given the complex nature of the wheat plot images in the test set. Somewhat surprisingly, however, our model struggles the most with images from the green spike, yellow canopy (GSYC) class, apparently as these images contained spikes that were the most challenging to detect.

**Table 3:** Count and evaluation of spike detection on test images from the SPIKE data set.

Image	GT	Det.	TP	FP	FN	Prec.	Recall	Accuracy	F1-Score
GSGC_test2	73	78	66	12	7	0.85	0.90	78%	0.87
GSGC test4	76	78	72	6	4	0.92	0.95	88%	0.94
GSGC test5	72	72	65	7	7	0.90	0.90	82%	0.90
GSYC test199	73	76	72	4	1	0.95	0.99	94%	0.97
GSYC test220	84	89	81	8	3	0.91	0.96	88%	0.94
GSYC test242	86	94	84	10	2	0.89	0.98	88%	0.93
GSYC test320	89	95	85	10	4	0.89	0.96	86%	0.92
GSYC test383	89	97	87	10	2	0.90	0.98	88%	0.94
GSYC test417	87	94	82	12	5	0.87	0.94	83%	0.91
GSYC test421	73	71	65	6	8	0.92	0.89	82%	0.90
GSYC test437	74	78	73	5	1	0.94	0.99	92%	0.96
GSYC test480	88	93	86	7	2	0.92	0.98	91%	0.95
YSYC test1	97	100	93	7	4	0.93	0.96	89%	0.94
YSYC test3	80	84	75	9	5	0.89	0.94	84%	0.91
YSYC_test6	102	100	91	9	11	0.91	0.89	82%	0.90
**Total**	1243	1299	1177	122	66	–	–	–	–
**Average**	–	–	–	–	–	0.91	0.95	86%	0.93
**SD**	9	10	9	2	3	0.03	0.03	0.04	0.03

**Figure 10:**
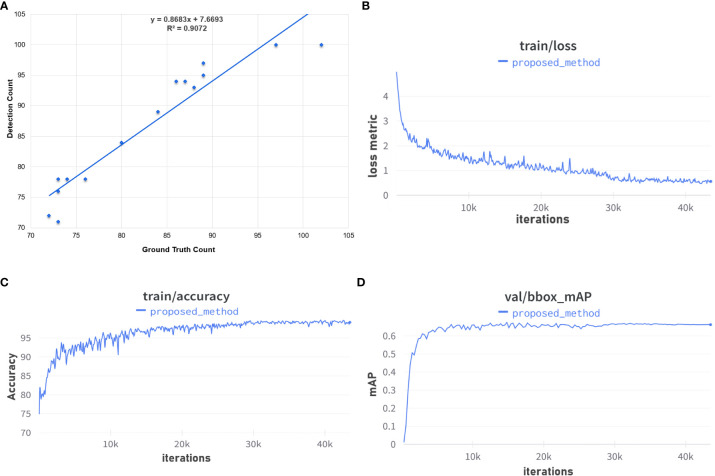
Overall performance measures: **(A)** the coefficient of determination; **(B)** the loss metric during training; **(C)** training accuracy; and **(D)** validation of training. **(A)** Ground Truth vs Detection Count Plot. **(B)** Training loss over 150 epochs. **(C)** Training accuracy over 150 epochs. **(D)** Validation set mAP over 150 epochs.

**Figure 10** summarizes the various accuracy measures employed to gauge performance. **Figure 10A** shows a plot of detected spikes as a function of actual count (ground truth). The slope of the line of best fit indicates that the model over estimates the true number of wheat spikes. Graphs in **Figures 10B**, **C** show the training losses and training accuracies, respectively, of our model over 150 epochs (43k iterations). It can be seen from the graphs that the training loss (shown in Equation 1) rapidly decreases over the first few epochs and then exhibits a more gradual decrease over the remainder of the training stage. Overall, the training loss of the model does not completely converge at the end of the training phase even though the training accuracy reaches its maximum value. Training accuracy reaches almost 100% after about 30k iterations of training, which coincides with the plateauing of the AP (at IoU = 0.5: 0.95: 0.05) value of the validation set (**Figure 10D**).

**Figure 11:**
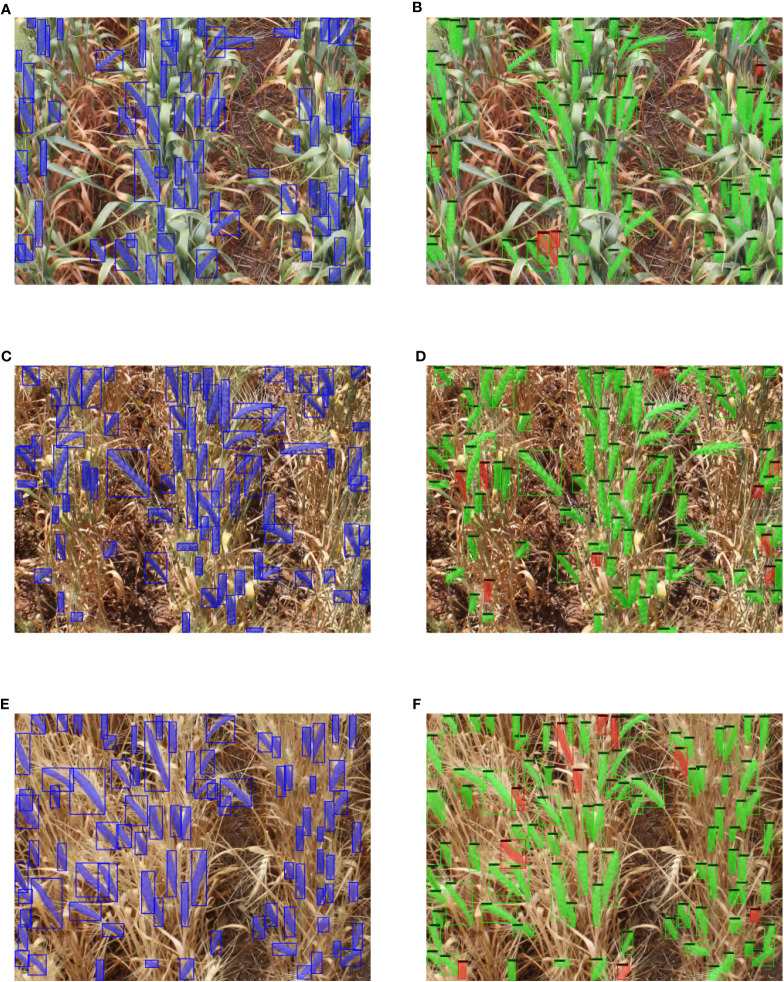
Visual example of ground truth vs detection results in test images of different growth stage. **(A)** GSGC ground truth image. **(B)** GSGC detection result. **(C)** GSYC ground truth image. **(D)** GSYC detection result. **(E)** YSYC ground truth image. **(F)** YSYC detection result.

In addition to these summary measures, we visualize in **Figure 11** typical detection and segmentation results of our model applied to the test set. The figure shows a comparison of our results with the ground truth masks in test images with complex background at different growth stages. The Blue bbox and masks represent ground truth annotations, Green bbox and masks represent true positive detections, and Red bbox and masks represent false positive detections.

**Figure 12:**
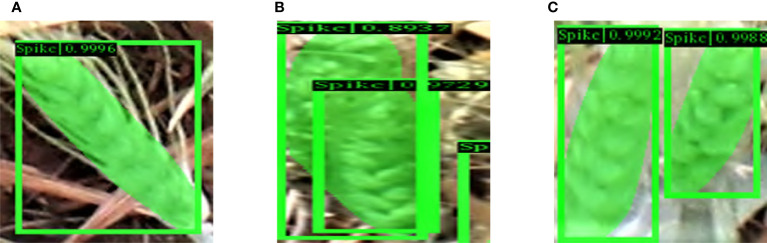
Examples of the different circumstances in which objects may appear in an image. **(A)** A completely visible spike, **(B)** Two overlapping spikes, **(C)** Partially visible spikes.

Some example cases of spike occlusion, such as when occluded by another spike or awns or leaves, are shown in **Figure 12**, which depicts the segmentation of spikes, where a mask within a yellow bounding box represents a detected spike (the color variations within the mask have no significant meaning and are only included to highlight the issue of occlusion). An example of a single spike, easily seen in **Figure 12A**, was reliably recognized by our model. Two overlapping spikes are visible in **Figure 12B**. The model is able to separate the two individual objects. Although it is challenging for any deep learning model to identify partially visible spikes, our model demonstrates a high success rate in such cases; partially visible instances of observed spikes are shown in **Figure 12C**.

### 4.2 Ablation analysis

We conducted an ablation analysis in which we omitted one or more of different components of the complete model architecture. The objective of this study was to assess the significance and quantitative impact of the model’s individual components. Such a study can assist in identifying the most important features of the model structure and provide some direction for future enhancements. During the study, we created different versions of the model with different combinations of the components. We refer to these versions as

1. Deformable Convolution Network (DCN) (Dai et al., 2017),
2. Generic RoI Extractor (GRoIE) (Rossi et al., 2021),
3. Side Aware Boundary Localization (SABL) (Wang et al., 2020), and
4. Auto-scaling LR (Goyal et al., 2017).

To evaluate the individual as well as combined impact of these components on the model’s performance, we set a baseline architecture based on a ResNet50 backbone, image scale = 1333 × 800, batch size = 2, and LR = 0.00125 (unless specified otherwise).

Not surprisingly, a comparison of these alternative approaches, shown in **Table 4**, reveals that the version involving all of the different components achieves the best AP at IoU = 0.5,0.93 and 0.94 for bbox and mask, respectively. However, the version not including Auto-scale LR and GRoIE, achieves the best AP for both IoU = 0.75 and IoU = 0.5: 0.95: 0.05 (shown in row 6). This is due to the fact that while GRoIE lets the model extract the RoI from all the levels of the Feature Pyramid from FPN, it does not guarantee the model will choose the RoI with the most accurate bounding box.

**Table 4:** Ablation analysis based on selective omission of one or more component of the proposed model.

DCN	GRoIE	SABL	AutoS LR	AP(IoU=0.5)		AP(IoU=0.75)		mAP (IoU=.5:.95:.05)		Epoch (optimal)	Train time (hr)	Infr. time (s/img)
													Bbox	Mask	Bbox	Mask	Bbox	Mask
–	–	–	–	0.9104	0.9155	0.7782	0.7841	0.6702	0.6268	222	10.5	0.77
✓	–	–	–	0.921	0.93	0.809	0.801	0.684	0.639	111	11	0.83
✓	✓	–	–	0.9177	0.925	0.796	0.806	0.66	0.6357	183	11	0.83
✓	✓	–	✓	0.9192	0.931	0.804	0.792	0.6781	0.6377	200	11.5	0.77
✓	–	✓	–	0.9195	0.9287	**0.8167**	**0.8162**	**0.689**	**0.6473**	103	12.5	0.83
✓	–	✓	✓	0.9212	0.9321	0.8024	0.7921	0.6712	0.6308	52*	11*	0.77*
✓	✓	✓	✓	**0.9303**	**0.9416**	0.8002	0.8009	0.6765	0.6436	70*	11*	0.87*

^*^ Training and testing of model was done using NVIDIA RTX 3080 12gb VRAM GPU. Bold value in a column represents the highest number (or, lowest for time).

Meanwhile, without GRoIE, the SABLHead can regress the bounding box from the RoI that is extracted from the top-most level of FPN thus getting bounding boxes with better IoU scores. This is reflected in the increased score of both AP (IoU = 0.75) and mAP metrics. Furthermore, with the help of GRoIE, SABLHead is able to regress bounding boxes from a larger number of samples or RoIs extracted. This led to more detected bounding boxes with an IoU score of at least 0.5, resulting in reduced false negatives and increased true positives, which is reflected in the higher value for AP (IoU = 0.5).

### 4.3 Quantitative comparison with other models

In order to get an overview of our model’s performance in comparison with other existing models, we conducted several experiments involving models with different backbones, LR schedulers and architectures. Our benchmarked scores are reported in **Table 5**. Different backbone networks showed different performances in our experiments.

**Table 5:** Comparative Analysis of models performance with different backbones, LR scheduler and architectures.

Type	Remark	AP (IoU=0.5)		AP (IoU=0.75)		mAP (IoU=.5:.95:.05)		Epoch (optimal)	Train time(hr)	Inference time(s/img)
		Bbox	Mask	Bbox	Mask	Bbox	Mask			
**ResNet50**	Backbone	0.917	0.9165	0.7797	0.7845	0.667	0.6278	230	10	0.67
**ResNet50 + DCN**	Backbone	0.93	0.9404	0.801	**0.8018**	**0.678**	**0.6459**	**51**	11	0.67
**MultiStep**	LR Scheduler	0.93	0.9404	0.801	**0.8018**	**0.678**	**0.6459**	**51**	11	0.67
**Linear Annealing**	LR Scheduler	**0.9303**	**0.9416**	0.8002	0.8009	0.6765	0.6436	70	11	0.87
**Faster RCNN**	Optimized	0.901	–	**0.8018**	–	0.669	–	241	**9**	**0.55**
**Mask RCNN**	Optimized	0.9047	0.9062	0.7459	0.7542	0.6327	0.6001	213	10	0.63
**Proposed**	–	**0.9303**	**0.9416**	0.8002	0.8009	0.6765	0.6436	70	**11**	**0.87**

* Training and testing of model was done using NVIDIA RTX 3080 12gb VRAM GPU. Bold value in a column represents the highest number (or, lowest for time).

ResNet50 with deformable convolution backbone achieved on average 1.42% and 1.99% better scores than the default ResNet50 backbone across all evaluation metrics for bbox and mask segmentation respectively. We also compared different learning rate schedulers to see which one would be better suited for our case. We reported the benchmark scores between Multistep and Linear Annealing learning rate schedulers. While Linear annealing showed a 0.3% and 0.12% increase in AP (IoU = 0.5), it was slower in terms of model convergence. So, we opted for using the Multistep LR scheduler throughout the rest of our experiments as it let the model converge to an optimal solution in less epochs than linear annealing LR scheduler. Model with LR scheduler also has around 23% slower inference speed compared to Multi-step LR scheduler, so there is a bit of tradeoff between precision and speed when choosing between different LR schedulers. We have made the decision to use the Linear Annealing Learning Rate (LR) scheduler in our model because it could produce better mAP scores (at IoU=0.5), but with a little trade-off in inference speed.

Finally, we compared different object detection and instance segmentation architectures, namely, Faster RCNN, Mask RCNN, and ours as in Cascade Mask RCNN, with a similar configuration of hyperparameters, to figure out the best architecture for our model. Our model with the Cascade Mask RCNN architecture outperforms all other methods by an average of 2.79% for bbox and 3.52% for the mask at AP (IoU = 0.5) while converging at only 70 epochs. Overall, we showed that our architecture and optimization strategy of choice provides a significant performance boost over all other compared architectures and strategies.

### 4.4 Comparison with existing approaches

In comparison to other existing methods Hasan et al. (Hasan et al., 2018), Wen et al. (Wen et al., 2022), Su et al. (Su et al., 2020) and Zhang et al. (Zhang et al., 2022) our model utilizing Cascade Mask RCNN architecture performs better in both bbox and mask segmentation. Our model was benchmarked across various scenarios, and several experiments involving models with different backbones, LR schedulers and architectures. For bounding box detection our model outperformed the best-performing existing model developed by Wen et al. and improved the AP(IoU=0.5) from 0.9262 to 0.9303 (increased by 0.41%). On the other hand, in terms of mask segmentation, our model out-performed the method developed by Zhang et al. and improved the AP (IoU=0.5) from 0.907 to 0.9416 (increased by 3.46%). Overall comparison with other state-of-the-art methods is shown in detail in **Table 6**.

**Table 6:** Comparison with other existing approaches.

Methods	AP (IoU=0.5)	
	Bbox	Mask
Hasan et al. (2019)	0.6763	–
Wen et al. (2022)	0.9262	–
Su et al. (2021)	0.567	0.572
Zhang et al. (2022)	0.904	0.907
Proposed *(WheatSpikeNet)*	0.9303	0.9416

**Figure 13:**
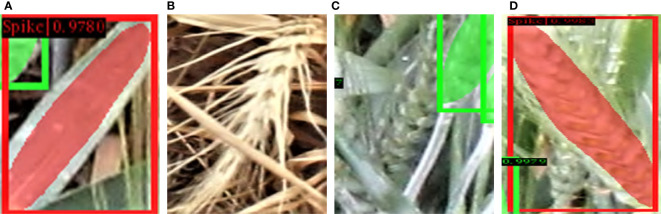
Limitations in the challenging environment. Here, Green bbox and mask denotes true positive detections, and Red bbox and mask denotes false positive detections. **(A)** ”Leaf” detected as ”spike”, **(B)** Partially visible ”spike” not detected, **(C)** Occluded ”spike” not detected, **(D)** Missed annotation correctly detected as "spike".

Our approach has shown some promising results in spike segmentation. However, there are a few limitations. When a leaf’s color is close to that of a spike, the model misidentifies the leaf as a spike, as seen in **Figure 13A**. In addition, there are a few spikes that the model overlooks due to a high density of spikes, and limited (partial) visibility of those spikes. **Figures 13B, C** both feature spikes that can be discerned by the human eye, but which the model is unable to identify. On the other hand, several spikes that were missed in the human annotation process were correctly identified as spikes by the model (**Figure 13D**), demonstrating the model’s efficacy. (Incidentally, it is worth remarking that the quantification of the model’s accuracy suffered as a result of these human annotation errors.)

## 5 Conclusion

This research addresses the phenotyping challenge of accurately segmenting wheat spikes from field images with high precision and persistence. Such images must be able to non-destructively discern, count, and segment wheat spikes in order to accurately estimate wheat production. This research concentrates on enhancing deep learning-based methods to handle the complexities of field images, such as illumination, occlusions, and various growth stages. The proposed method introduces a novel architecture for the spike segmentation task which differs significantly from existing methods since for accurate detection and segmentation of the spike regions we fused the Cascade Mask RCNN with other extra precising techniques of DCN, GRoIE, SABL and Auto-scaling LR. Several iterations of repeated trials were applied to fine-tune and optimize the architecture and hyper-parameters of the model to satisfy the criteria. Along with a few limitations such as human annotation error, misidentifies the leaf as a spike, overlooking due to a high density and limited visibility of spikes; the approach offers a significant improvement over existing techniques, which have hitherto been recognized as state-of-the-art, such as Zhang et al.(Zhang et al., 2022), Su et al. (Su et al., 2020), Wen et al.(Wen et al., 2022) and Hasan et al. (Hasan et al., 2018). This improvement enhances the capability to segment and count spikes in images captured under challenging field conditions, such as variable illumination, shadowing, or high congestion. The approach is applicable to a wide variety of complex real-world situations, in part because it employs a flexible data set compiled from land-based field imaging under real-world conditions. In addition, the enhanced model can be deployed with various imaging modalities, including UAVs and possibly satellites. The precise segmentation and counting of multiple phenotypic characteristics, such as wheat spikes and spikelets, paddy and sorghum head, allows for more precise crop breeding and management decisions. The research findings presented in this article represent a significant step towards the realization of the promise of e-agriculture, specifically AI, as an instrument for enhancing agricultural productivity.

## Data availability statement

The codebase developed and the datasets presented in this study can be found in online repositories. The names of the repository/repositories and accession number(s) can be found below: https://figshare.com/projects/WheatSpikeNet_An_Improved_Wheat_Spike_Segmentation_Model_for_Accurate_Counting_from_Field_Imaging/163225.

## Author contributions

MMH and SJM contributed to conception and design of the study. MMH, MAB, and MI organized the database. MAB, and MI performed the statistical analysis. MAB and MI wrote the first draft of the manuscript. AKMA, MMH, and MAH contributed to the visualization and representations in the draft. MMH, SJM, MAB, and MI wrote sections of the manuscript. MMH, SAA, AKMA, and SJM proofread the manuscript for final submission. MMH, and SJM supervised the project. MAH, and SAA contributed in project administration. All authors contributed to manuscript revision, read, and approved the submitted version.
